# Multiplex Assay for Simultaneous Detection of *Mycoplasma genitalium* and Macrolide Resistance Using PlexZyme and PlexPrime Technology

**DOI:** 10.1371/journal.pone.0156740

**Published:** 2016-06-06

**Authors:** Sepehr N. Tabrizi, Lit Y. Tan, Samantha Walker, Jimmy Twin, Marin Poljak, Catriona S. Bradshaw, Christopher K. Fairley, Melanie Bissessor, Elisa Mokany, Alison V. Todd, Suzanne M. Garland

**Affiliations:** 1 Department of Microbiology and Infectious Diseases, The Royal Women’s Hospital, Parkville, Victoria, Australia; 2 Department of Obstetrics and Gynecology, University of Melbourne, Victoria, Australia; 3 Murdoch Childrens Research Institute, Parkville, Victoria, Australia; 4 SpeeDx Pty Ltd, Sydney, NSW, Australia; 5 Melbourne Sexual Health Centre, Carlton, Victoria, Australia; 6 Central Clinical School, Monash University, Victoria, Australia; Miami University, UNITED STATES

## Abstract

*Mycoplasma genitalium* is a cause of non-gonoccocal urethritis (NGU) in men and cervicitis and pelvic inflammatory disease in women. Recent international data also indicated that the first line treatment, 1 gram stat azithromycin therapy, for *M*. *genitalium* is becoming less effective, with the corresponding emergence of macrolide resistant strains. Increasing failure rates of azithromycin for *M*. *genitalium* has significant implications for the presumptive treatment of NGU and international clinical treatment guidelines. Assays able to predict macrolide resistance along with detection of *M*. *genitalium* will be useful to enable appropriate selection of antimicrobials to which the organism is susceptible and facilitate high levels of rapid cure. One such assay recently developed is the MG 23S assay, which employs novel PlexZyme™ and PlexPrime™ technology. It is a multiplex assay for detection of *M*. *genitalium* and 5 mutations associated with macrolide resistance. The assay was evaluated in 400 samples from 254 (186 males and 68 females) consecutively infected participants, undergoing tests of cure. Using the MG 23S assay, 83% (331/440) of samples were positive, with 56% of positives carrying a macrolide resistance mutation. Comparison of the MG 23S assay to a reference qPCR method for *M*. *genitalium* detection and high resolution melt analysis (HRMA) and sequencing for detection of macrolide resistance mutations, resulted in a sensitivity and specificity for *M*. *genitalium* detection and for macrolide resistance of 99.1/98.5% and 97.4/100%, respectively. The MG 23S assay provides a considerable advantage in clinical settings through combined diagnosis and detection of macrolide resistance.

## Introduction

*Mycoplasma genitalium* was first isolated in 1980 from men with non-gonococcal urethritis (NGU) [1]. It has since been established as a common sexually transmitted infection responsible for 10–35% of non-chlamydial NGU in men [2] and also associated with cervicitis, endometritis, pelvic inflammatory disease (PID) and tubal factor infertility in women [3–7]. Studies also suggest *M*. *genitalium* plays an important role in HIV acquisition and transmission [8, 9].

Current recommended treatment for uncomplicated *M*. *genitalium* infection is a single 1 g oral dose of the macrolide antibiotic, azithromycin [10, 11]. In most developed countries, this is also the first line treatment for NGU. However cure rates following this treatment have declined to about 70% for genital *M*. *genitalium* infections, as macrolide resistance has emerged [12–14] [15].

Macrolide resistance occurs through mutations in nucleotide positions 2058 and 2059 (*E*.*coli* numbering) in region V of the 23S rRNA gene, inhibiting binding of the macrolide to *M*. *genitalium* [14, 16]. An important limitation in current clinical practice is the ability to effectively treat this organism in a significant proportion of individuals, when prevalence of resistance to first line therapies is so high. To address this, a high resolution melt analysis (HRMA) methodology was recently described to rapidly detect 23S rRNA gene mutations associated with macrolide resistance in *M*. *genitalium* positive specimens [17]. This HRMA assay allowed for screening of 23S rRNA mutations; however it needs to be performed as a separate assay after *M*. *genitalium* detection, delaying results, second line treatment and potentiating further transmission.

Development of an assay for the simultaneous, rapid and sensitive detection of *M*. *genitalium* and mutations associated with macrolide resistance would offer an enormous clinical advantage, facilitating rapid delivery of the most appropriate therapy. This study evaluates the MG 23S assay, which employs novel PlexZyme™ and PlexPrime™ technology (SpeeDx), is a single well, multiplex qPCR assay, which simultaneously detects *M*. *genitalium* and five 23S rRNA mutations associated with macrolide resistance.

## Methods

### Patient population

The assay was evaluated using clinical samples previously screened for both *M*. *genitalium* detection by 16S rRNA qPCR and 23S rRNA mutation status by HRMA and Sanger sequencing. Between July 2012-June 2013, 400 specimens (including baseline, day 14 and/or day 28 specimens) from 254 consecutive *M*. *genitalium* infected participants (186 males (300 urine/urethral and 12 anal samples) and 68 females (41 urine, 42 cervical/vaginal and 5 anal samples)) were collected at Melbourne Sexual Health Centre, Victoria, Australia. This clinic conducts routine testing for *M*. *genitalium* in patients with NGU, cervicitis, and/or PID, as well as sexual contacts of *M*. *genitalium* infected patients. Ethical approval for this study was granted by the Alfred Hospital Research Ethics Committee (number 150/12).

### Assay description

**Fig 1:**
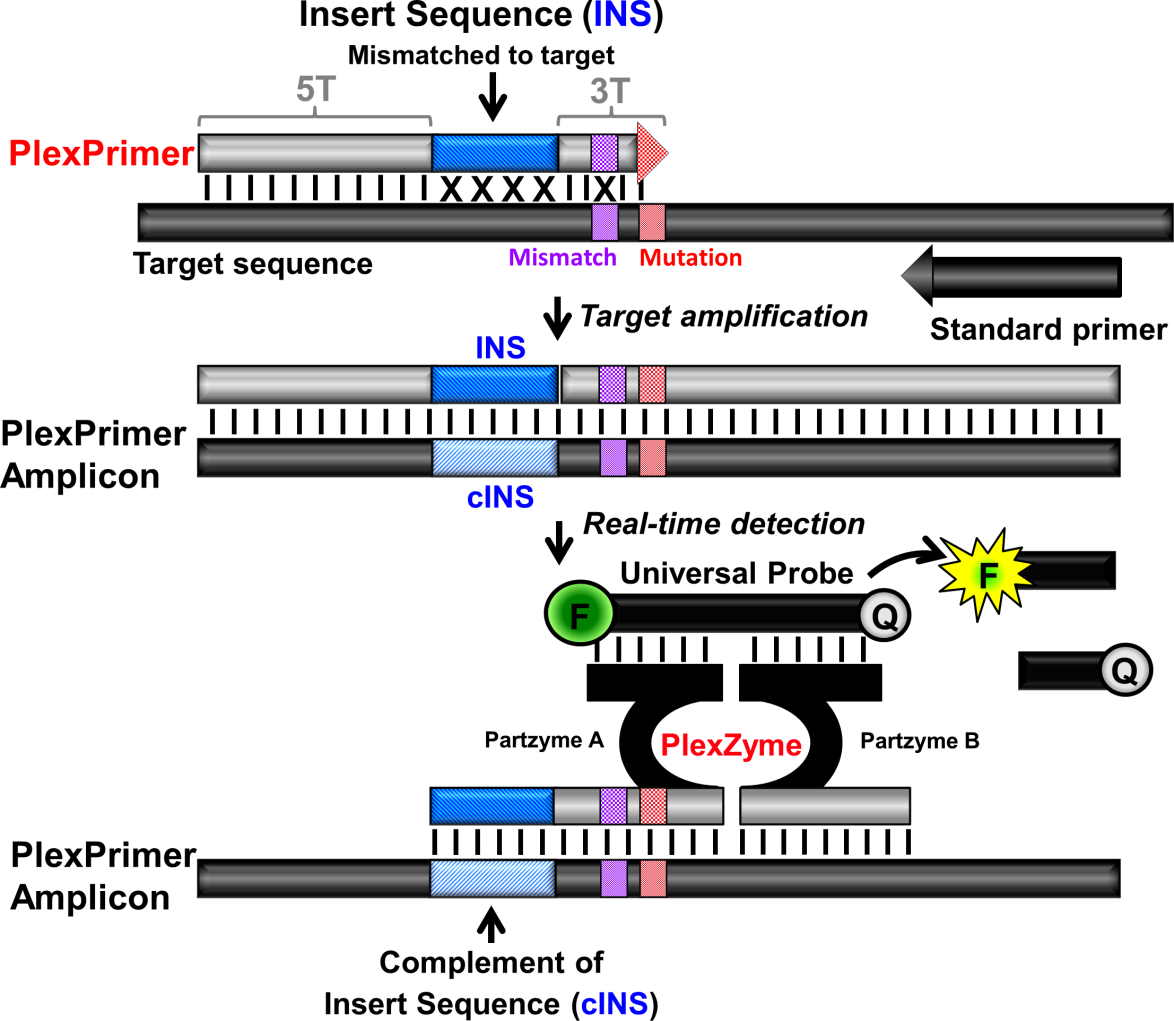
PlexPrimer and PlexZyme detection technology. The Plexprimer contains three functional regions; a 5’ target recognition region (5T), a short 3’ target-specific sequence (3T) and an intervening Insert Sequence (INS) region, which is mismatched with respect to the target. The PlexPrimer binds to the mutation at the 3’ terminus (red box) and also contains a mismatched base (purple box). During amplification the INS and its complement (cINS) are incorporated into the PlexPrime amplicons and these can be detected in real-time using PlexZymes. PlexZymes are nucleic acid enzymes which only form, from their component partzymes A and B, when target amplicon are present. Each of the partzymes contain a probe binding arm, a partial catalytic core and a target binding arm, orientated such that partzyme A binds to the amplicon in the region containing the cINS whilst the Partzyme B binds adjacently downstream. Catalytically active PlexZymes bind and cleave universal reporter probes between fluorophore (F) and quencher (Q) moieties resulting in signal generation.

The MG 23S assay (SpeeDx Pty Ltd, Sydney, Australia) is a multiplex qPCR assay detecting across three fluorophore channels: 1) FAM (495–516) for detection of *M*. *genitalium* through detection of the *MgPa* gene, 2) Hex (535–556) for detection of 5 mutations that have been described in clinical samples (A2058G, A2059G, A2058C, A2059C and A2058T) in the 23S rRNA gene and 3) Cy5 (650–670) for detection of a heterologous extrinsic control to monitor extraction efficiency and qPCR inhibition. This assay employs novel PlexZyme (formerly known as MNAzyme) technology [18–20] and PlexPrimers which selectively amplify target sequences, coupled to enable high level multiplexing of mutant detection (Fig 1).

### Template and Targets

Quantified synthetic DNA templates from the MG 23S Positive Control (beta version) (SpeeDx) were utilized for the limit of detection testing of *M*. *genitalium* (*MgPa* gene) and mutations from the 23S rRNA gene; A2058G, A2059G, A2058C, A2059C and A2058T. Testing of cross-reactivity of the MG 23S assay was performed with 10^4^ copies of Amplirun^®^ DNA (Vircell, Granada, Spain) for *Mycoplasma hominis* (Cat# MBC084), *Trichomonas vaginalis* (Cat# MBC079), *Chlamydia trachomatis* (Cat# MBC012), *Neisseria gonorrhoeae* (Cat# MBC075) and *Mycoplasma pneumoniae* (Cat# MBC035), and 10^5^ copies of extracted DNA from clinical isolates of *Ureaplasma parvum* and *Ureaplasma urealyticum*. We have further tested extracted DNA from clinical isolates of further 24 unrelated species including *Salmonella typhimurium*, *Klebsiella pneumoniae*, *Haemophilus influenzae*, *Enterococcus faecalis*, *Proteus mirabilis*, *Staphylococcus aureus*, *Staphylococcus epidermidis*, *Candida albicans*, *Streptococcus pyogenes*, *Pseudomonas aeruginosa*, *Acinetobacter calcoaceticus*, *Neisseria gonorrhoeae*, *Bacteroides fragilis*, *E*. *coli*, Viridans streptococci, Streptococcus Group A and B, *Campylobacter jejuni*, *Neisseria meningitidis*, *Neisseria cinerea*, *Neisseria subflava*, *Neisseria lactamica*, *Chlamydophila pneumoniae* and *Chlamydia trachomatis*.

### Analytical Sensitivity

The sensitivity of each of the targets of the MG 23S assay was determined as the lowest number of synthetic DNA template copies tested with ≥95% detection.

### Processing and PCR

Samples were processed and extracted as described previously [12]. The reference method for detection of *M*. *genitalium* was an established qPCR assay targeting the 16S rRNA gene [21]. The reference standard for determining the presence of a mutation was HRMA and if any were unable to be determined using this method, the samples underwent sequencing of the region V of 23S rRNA as described previously [16, 17]. The MG 23S assay was performed as described by the manufacturer. Briefly, a 5 μl aliquot of the extracted DNA was mixed with Plex Mastermix in 20 μl final volume. All testing was performed in 96 well plates on the LightCycler^®^ 480 Instrument II (Roche Diagnostic, Indianapolis, USA) using parameters of 95°C for 2 min, followed by 10 cycles of 95°C for 5 s, 61°C for 30 s (-0.5°C per cycle), and 40 cycles of 95°C for 5 s, 52°C for 40 s. Data analysis was performed using an analysis algorithm provided with the assay, resulting in reporting of the presence or absence of *M*. *genitalium*, any of the five mutations in the 23S rRNA gene, and the internal control. Controls for the assay were comprised of synthetic double stranded DNA targets for each 23S rRNA gene mutation type and *M*. *genitalium* G37 genomic DNA (ATCC 33530D) serving as the wild type and *M*. *genitalium* control.

## Results

The limit of detection for the *M*. *genitalium* MgPa target was 10 copies. The limit of detection of 23S rRNA gene targets was 10 copies for A2058C, A2058T and A2059G, 12 copies for A2058G, and 15 copies for A2059C (Table 1). Cross-reactivity of primer and probe sequences for both MgPa and 23S rRNA were first analyzed *in silico* using BLAST to ensure specificity. Subsequent testing of the assay using DNA from related organisms and other clinically relevant organisms showed no cross-reacting agents for the MgPa assay or the 23S rRNA mutant assay.

**Table 1:** Analytical sensitivity of the MG 23S assay as determined by detection of replicate of different concentration of each template.

	Copy number Detected/replicate tested						Copy number (detected/ replicates tested)
Template	10^5^	10^4^	10^3^	10^2^	10	1	
**A2058G**	3/3	3/3	3/3	3/3	3/3	2/3	12 (20/20)
**A2058C**	3/3	3/3	3/3	3/3	3/3	3/3	10 (20/20)
**A2058T**	3/3	3/3	3/3	3/3	3/3	1/3	10 (19/20)
**A2059G**	3/3	3/3	3/3	3/3	3/3	1/3	12 (20/20)
**A2059C**	3/3	3/3	3/3	3/3	2/3	0/3	15 (20/20)
**MgPa**	3/3	3/3	3/3	3/3	3/3	2/3	10 (20/20)

Detection of *M*. *genitalium* by the MG 23S assay was highly concordant (99.0%) with the reference method (qPCR targeting the 16S rRNA gene) with a Kappa value of 0.965 (95% confidence interval (CI) 0.930 to 0.999) and sensitivity/specificity of 99.1% and 98.5% respectively (Table 2). Only 4 (1.0%) of the specimens were discordant with one positive only by the MG 23S assay and 3 positive only by the reference assay. All of the discordant samples had low copy number of *M*. *genitalium*. Among the 330 specimens positive for *M*. *genitalium*, 325 (98.5%) were concordant for detection of a macrolide mutation, with a Kappa value of 0.969 (95% CI 0.942–0.996) and sensitivity/specificity of 97.4% and 100% respectively (Table 3). Only 5 cases with macrolide mutations detected by the reference method were classified as wild type by the MG 23S assay, and there were no cases with macrolide resistance detected by the MG 23S assay, where the reference method had shown a wild type status.

**Table 2:** Evaluation of the MG 23S assay for the detection of *M*. *genitaliu*m.

		Reference Assay^a^			
		Pos	Neg	Total N (%)	Sensitivity % (95% CI) / Specificity % (95% CI)
PlexPCR	Pos	330	1	331 (83)	99.1 (97.4–99.8) / 98.5 (92.0–100.0)
	Neg	3	66	69 (17)	
	Total	333	67	400	

^a^ The reference assay for *M*. *genitalium* detection was an in house qPCR assay targeting the 16S rRNA gene [21].

**Table 3:** Evaluation of the MG 23S assay for the detection of 23S rRNA mutations.

		Reference Assay^a^			
		MT^b^	WT^b^	Total N (%)	Sensitivity % (95% CI) / Specificity % (95% CI)
PlexPCR	MT^b^	184	0	184 (56)	97.4 (93.9–99.1) / 100.0 (97.4–100.0)
	WT^b^	5	141	146 (44)	
	Total	189	141	330^c^	

^a^ The reference assay for 23S mutation status was HRMA and Sanger sequencing [16, 17].

^b^ MT refers to 5 common mutation detection of adenine to another base in 2058 or 2059 position of 23S rRNA. WT refers to adenine in both 2058 and 2059 positions of 23S rRNA.

^c^ Only includes *M*. *genitalium* positive samples determined by reference method and excludes three samples in which 23S sequence status could not be confirmed.

## Discussion

Overall, the MG 23S assay showed excellent performance for the simultaneous detection of *M*. *genitalium* and 23S rRNA mutations by qPCR, with high sensitivity and specificity compared to established gold standards.

Macrolide resistance is highly prevalent in *M*. *genitalium* and exceeds 30% in many countries. As culture is difficult and rarely performed to allow performance of susceptibility testing, a molecular approach has been utilized to predict resistance to azithromycin. All azithromycin treatment failures to date have been shown to carry one of the five mutations in the 2058 and 2059 positions in the 23S rRNA gene [12], therefore detection of these mutations can be used to infer macrolide resistance. Molecular methodologies such as pyrosequencing, HRMA or FRET methods [17, 22] have been used to detect the presence of macrolide resistance mutations [23]. However these assays must be performed after the initial *M*. *genitalium* diagnostic test, delaying reporting of the mutation status. Other qPCR technologies are able to detect mutations such as mutation-specific primers and mutation-specific probes [24–26]. However, it is difficult to efficiently multiplex mutation-specific primers as they may only differ by a single base. Mutation-specific probes are also similarly limited in multiplexing due to competition. The novel design of the PlexPrimer overcomes this limitation by incorporating a unique sequence into the amplicon, to increase differentiation and reduce competition. PlexPrimers coupled with PlexZymes enables high level multiplexing, for specific mutation amplification and detection in qPCR.

The implementation of combined diagnostic and resistance testing to optimize rapid selection of more effective antimicrobials is considered a priority in the field. This would help to reduce recurrent clinical presentations to services, sequelae such as infertility, and secondary transmission of resistant strains. The MG 23S assay offers an enormous advantage in clinical settings, as it can be incorporated into clinical algorithms. Detection of 23S rRNA mutations would allow patients to be rapidly treated with an appropriate second line antibiotic such as the fluoroquinolone moxifloxacin, rather than waiting 3–4 weeks for a test of cure to show azithromycin treatment failure. Patients without evidence of macrolide resistance can currently be treated with azithromycin with high cure rates. There are however, up to 10% of patients that do not have detectable pre-treatment mutations, and appear to develop induced or selected resistance following azithromycin treatment. Treatment failure in these cases will not be avoided by the use of pre-treatment resistance assays. This will only be averted by the use of non-macrolide first line regimens; however identification of suitable alternatives has proven difficult. In addition to macrolides, *M*. *genitalium* is also developing resistance to moxifloxacin [12]. Development and inclusion of further markers for fluoroquinolone resistance would be greatly beneficial and is currently under development.
